# Assessment of community pharmacy professionals’ knowledge and counseling skills achievement towards headache management: a cross-sectional and simulated-client based mixed study

**DOI:** 10.1186/s10194-018-0930-7

**Published:** 2018-10-16

**Authors:** Adeladlew Kassie Netere, Daniel Asfaw Erku, Ashenafi Kibret Sendekie, Eyob Alemayehu Gebreyohannes, Niguse Yigzaw Muluneh, Sewunet Admasu Belachew

**Affiliations:** 1 Department of Clinical Pharmacy, School of Pharmacy, College of Medicine and Health Sciences, University of Gondar, P.O. Box: 196, Chechela Street, Lideta Sub City Kebele 16, Gondar, Ethiopia; 2 Department of Psychiatry, University of Gondar, Chechela Street, Lideta Sub City Kebele 16, Gondar, Ethiopia

**Keywords:** Community medicine retail outlets, Counseling, Headache, Pseudo client, Ethiopia

## Abstract

**Background:**

Headache is one of the most common disabling medical condition affecting over 40% of adults globally. Many patients with headache prefer to alleviate their symptom with a range of over-the-counter analgesics that are available in community medicine retail outlets (CMROs). However, data regarding how community pharmacists respond to headache presentation and their analgesic dispensing behaviors in Ethiopia is scarce. The present study aimed to assess the self-reported and actual practice of community pharmacists toward management of a headache in Gondar town, Ethiopia.

**Methods:**

A dual-phase mixed-methods research design, including pseudo-client visits (between April 1 and 30, 2018) followed by a questionnaire-based cross-sectional study (between May 1 and 20, 2018) was conducted among CMROs in Gondar town, Ethiopia.

**Results:**

Among the 60 pseudo-client visits, 95% of them dispensed medications. The overall counseling approach was found to be 42.6% which improved to 58.3% when the pseudo-clients demanded it. Duration (73.3%) and signs/symptoms (45%) of headache were asked before dispensing the medications. Dosing frequency (86.7%), indication (60%) and dosage form (35%) were the most discussed items. Ibuprofen (45%) and diclofenac (41.5%) were primarily added to paracetamol for better headache treatment. Effectiveness (61.7%) and cost (21.7%) were the main criteria to choose drugs. In the cross-sectional survey, 60 participants were requested and 51 of them agreed to participate (response rate of 85%). Of these participants, 64.7% agreed that managing headache symptomatically is challenging. Patient lack of confidence in dispensers (41.2%) and lack of updated medical information (31.4%) were reported as the primary barriers to counsel clients.

**Conclusion:**

This study demonstrated the practical gaps in counseling practices and poor headache management of community pharmacies in Gondar city. National stakeholders in collaboration with academic organizations should be involved in continuous clinical training and education regarding proper counseling practices.

## Background

Headache or cephalalgia affects infrequently almost everyone [1]. The Global Burden of Disease Study 2015 (GBD2015) ranked migraine as the third highest cause for disability worldwide in persons younger than 50 years in both sexes [2]. and nearly 40% of people suffered from a headache at some time in their lives [1]. Around 15% of UK adult patients experience a migraine with a three-to-one ratio of women-to-men [1]. According to a population-based national survey of headache burdens in Ethiopia, the prevalence had been reported as migraine (17.7%), tension-type headache (TTH) (20.6%), probable medication-overuse headache (pMOH), and headache yesterday 6.4% [3]. Patients most regularly seek professional counseling from general and neurologic clinics [1, 4]. Headache challenges healthcare professionals in many ways and represents enormous social and economic burden to the health care system.; for example, 20 billion USD is lost every year in the United States for migraine [1, 2, 5].

Community pharmacists are the most accessible healthcare professionals to the public owing to their convenient location at the heart of the community and wide geographic distribution [6]. Patients with a headache successfully self-medicate by means of over-the-counter (OTC) medications that are available via CMROs [7]. This provides a unique opportunity for pharmacy staffs to play a crucial role in ensuring the quality use of medications by providing patients with counseling on the safe, correct and effective use of medicines, and solving potential drug-related problems [8, 9]. Even though CMROs can sufficiently treat minor ailments and contribute in self-care management such as headaches, still they need to be careful while recommending OTC drugs since even these drugs can cause health threats if used inappropriately [10]. Findings from developing countries showed that dispensers working in the pharmacies hardly keep sufficient knowledge and skills for effective syndrome management [11]. On the other hand, pharmacists in developed nations, such as the United Kingdom and Australia, successfully incorporated minor ailment management with other public-health programs [12–15]. In Ethiopia as in most developing countries pharmacy staff are largely confined to the traditional medication dispensing and counseling practices and once in a while delivering such public health services [16, 17] which is worsened by the absence of standard and consistence treatment (counseling) guidelines for headache and other common minor ailments [18]. Regarding self-medications and related issues, a variety of investigations were conducted in many parts of Ethiopia, though many of them used client perceptions [19, 20]. Owing to the burden of headache in Ethiopia [3], patients usually look for immediate therapy in the nearby public pharmacies. A recent study conducted by Ayele et al. identified lack of access to clinical training and poor community awareness as the most commonly cited barriers for providing public health services in CMROs such as headache management [21]. Yet, the extent to which pharmacy professionals interacts with patients for headache management is not studied in detail, and there is no published data that explores pharmacy staff knowledge and counselling skills when it comes to handling patients’ request of analgesic medications. Thus, the current study aimed at assessing the knowledge and extent of community pharmacy professionals’ involvement in counseling practices and overall management of a headache as well as to explore the challenges and hidden reasons that hindered professionals from delivering the standard care to the clients.

## Methods

### Study design and setting

A dual-phase mixed-methods research design, including a simulated client method (April, 2018) and a cross-sectional study survey (May, 2018) were employed. The study was conducted in Gondar town, Northwest Ethiopia. The town has a population of approximately 206,987 [22] and 66 community medicine retail outlets (CMROs) (40 community pharmacies and 26 drug stores). This study was reviewed and ethically approved by the Institutional Review committee of the University of Gondar, School of Pharmacy with the approval number of (UOG-SOP204/2018). The data collected were kept anonymous and no personal identifier were used.

### The simulated patient (SP) study

According to Food, Medicine and Healthcare Administration and Control Authority of Ethiopia (FMHACA), community medicine retail outlets (CMROs) are mainly classified into a pharmacy, drug store (shop) and drug vendor. Gondar town’s CMROs were classified by geographical locations such as Arada, Piassa, Lideta (Chechela), Maraki and Azezo sub-cities (Kifle Ketemas) and all of the community medicine retail outlets were taken as study samples. Each of the CMROs was visited once by a pseudo-patient within study period giving a total of 60 (1 in each CMROs) pseudo-patient visits. Details of the scenario (an intermittent headache) employed in the simulated study are presented in Table 1. The pharmacist was expected to rule out other medical conditions, medication history and advise the pseudo-client to take paracetamol combined with other analgesics such as nonsteroidal anti-inflammatory drugs (NSAIDS) or weak opioids like tramadol, and if inadequate and the symptom still persists advice to visit the nearby hospital or clinic.

**Table 1:** The scenario employed in the simulated study, Gondar, 2018

Intermittent headache	
The SP is a 20-year-old male with a complaint of an intermittent (moderate–to-severe) headache for 04 days duration. The SP is currently taking paracetamol to alleviate his symptom. Yet, he sensed that he needs a more effective treatment and, hence, visited a community pharmacy.	
The pharmacist was given the following information when asked:	The SP had no other previous or current medical condition. The SP did not drink coffee and alcohol and was non-smoker.
	The headache started 04 days back and the SP had the symptom for most of the days.
	The pain was described as mild, dull, low intensity, and affecting both sides of the head.
	Paracetamol was the only medication the SP was taking during that time.
	There were no special factors that trigger/worsen the headache.
	The patient did not visit a hospital for this cause.

### The pseudo-client method

Pseudo (simulated)-patient method is a technique of assessing the service providers’ (dispensers’) counseling practices in CMROs by visiting the pharmacy staff after training specific patient scenario. The aim of the pseudo-client-based study was to assess the participation of CMROs dispenser staffs in the management of a self-diagnosed headache and to explore the challenges and hidden reasons that impede the delivery of standard counseling services to the clients. This method has been employed extensively and comprehensively in pharmacy practice-based researches [23, 24].

Two clinical pharmacists acted as simulated patients. A half-day long discussion and training were given to the SPs so that they will be familiar and be able to perform the given clinical scenario. They were instructed not to give and/or ask further information unless asked by the pharmacy staff so as to make sure that the information provided by each SPs is uniform across all visits. In order to avoid dependence on the human cognitive processes, which has been mentioned as a potential limitation of the simulated patient method [25], all the visits were audio recorded. Immediately after each visit, the SPs filled the data gathered into a form containing a checklist of items (such as enquiries including: client history, information provided by dispensers, self-diagnosis, medication selection process, direction for use and medical profile, aggravating and alleviating factors, and duration of headache) that were intended to assess the practice of pharmacy personnel toward the dispensing of antibiotics for the specified minor ailments. The principal investigator (AKN) compared and validated the data from the checklist against audio recordings for the purpose of quality assurance.

### The cross-sectional study

A self-administered English version questionnaire was prepared and distributed to 63 community pharmacists (one in each CMRO) after securing verbal and written consent and clarification of the aim of the study. One of the working staffs of dispenser was selected randomly when there were two or more dispenser staffs during data collection. The questionnaire contains respondents’ demographic factors, working hours, average client waiting time, dispensing experiences, headache management practices, and potential barriers to proper service delivery. Each questionnaire took an average of 15 min. Finally, the completed questionnaire was collected on-site by the investigators.

### Data quality control, entry, analysis and interpretation

The overall data was checked for its completeness and accuracy and important variables were addressed. Data from both the simulated and the cross-sectional studies were entered into and analyzed using Statistical Package for Social Studies (SPSS) version 20.0 [26]. The results were presented as frequencies and percentages.

### Operational definitions

Community medicine retail outlets in our study as described in [27]:

#### Pharmacy

It denotes a drug shop having the mandate to hold any medicine and medical equipment. In addition, the professional who is supposed to dispense inside the pharmacy is ‘A pharmacist’. No one else is allowed to dispense according to the FMHACA of Ethiopia.

#### Drug store

Unlike a pharmacy, a drug store is a drug shop but the medicine to be dispensed here is restricted. That means, it is not legal to hold every medication in this medicine retail outlet. For instance: It is not allowed to hold medications like psychotropic/narcotic drugs. In addition, the professional who is supposed to dispense inside the drug store is ‘A druggist’.

#### Pharmacists

In Ethiopia, pharmacists are professionals having a bachelor degree from private or government university. In addition, they took all the courses that one medication expert has to know at the end. The duration of the study to be a pharmacist used to be four years which has been changed to five years since 2008.

#### Druggists

We can use this name interchangeably with’ Pharmacy technician’. In Ethiopia, those professionals having ‘diploma degree’ from colleges are considered druggists (i.e. it is not a university-level education). They took courses for 3 years only and it is not that much comprehensive like pharmacists..

## Result

### Pseudo client approach

Out of the 66 CMROs, 60 of them were visited by the pseudo-patient approach whereas the rest were non-functional (closed) during the data collection. Among the evaluated respondents, the majority (*n* = 32, 53.3%) were females. Thirty-four (56.7%) of the premises were leveled as pharmacies. Almost all the participants dispensed medications for the pseudo-patient (95%), while 3 (5%) dispensers suggested the client to consult physicians to identify the cause of a headache. More than half of the participants provided information when the pseudo-client demanded it (58.3%) and dispensed generic drugs (73.3%); ibuprofen (45%) and diclofenac (41.5%) were the most recommended ones for a headache in addition to paracetamol. Effectiveness (61.7%) and cost (21.7%) were the major reasons to choose drugs for pseudo-clients as illustrated in Table 2.

**Table 2:** Drug selections and dispensing practices of CMROs in Gondar city during pseudo-client

Variables	*N* (%)
Gender	
Male	28 (46.7)
Female	32 (53.3)
Community medicine retail outlets level	
Pharmacy	34 (56.7)
Drug store	26 (43.3)
Dispensers who dispensed the drug for pseudo patient	57 (95)
Dispensers send SP to consult the doctor; not dispensed drugs	3 (5)
Dispensed drugs product based on name	
Generic	44 (73.3)
Brand	13 (21.7)
Drugs were selected based on	
Effectiveness	37 (61.7)
Cost	13 (21.7)
Both effectiveness and cost	7 (11.7)
ADR	0
Availability	0
Type of drug added to PCM for headache management	
Diclofenac	25 (41.7)
Ibuprofen	27 (45)
Tramadol	3 (5)
ASA	0
Acetaminophen with tramadol combined formulation	2 (3.3)
Provision of information for the pseudo client approach	
Spontaneously	22 (36.7)
Enquired about sign and symptoms	35 (58.3)

Approach, 2018 (*N* = 60)

Overall professional counseling approach of the dispensers was assessed by a five-point Likert scale (poor = 1, fair = 2, good = 3, very good = 4, and excellent = 5). The mean (±SD) counseling approach of dispensers was 2.14 (0.9) which means only 42.6% of dispensers gave proper counseling. During the pseudo-client approach, most dispensers asked primarily duration (*n* = 44, 73.3%) and types of signs/symptoms (*n* = 27, 45%) of a headache before dispensed the medications. Also, 48.3% of them allowed SP to be involved in the medication-selection process. Just below half (45%) of the providers inquired signs or symptoms and around 22% asked the SPs about the type of medication previously taken. Only 5 (8.3%) dispensers asked whether they have previous or current medical condition whereas nearly 12% of them asked about their current medication profile other than paracetamol. Furthermore nobody asked about whether the pseudo-client needed additional information, the presence of allergic history, adverse drug reaction, and alleviating factors (Table 3).

**Table 3:** Questions and patient history’s required by the provider during encounter

Items	Responses	
	Yes (%)	No (%)
SPs age was asked during dispensing	6 (10)	54 (90)
The provider let SP to be involved in the medication-selection process	29 (48.3)	28 (46.7)
The provider asked whether SP needed additional information	0	57 (95)
The provider personnel asked about the presence of specific conditions that could affect diagnosis or recommended treatment	3 (5)	54 (90)
Starting time of headache	13 (21.7)	44 (73.3)
Location of pain	1 (1.7)	56 (93.3)
Magnitude (intensity) of pain	4 (6.7)	53 (88.3)
Duration of headache asked	44 (73.3)	13 (21.7)
Types of typical signs/symptoms of headache asked	27 (45)	30 (50)
Current medication other than PCM asked	7 (11.7)	50 (83.3)
Previous or current medical condition asked	5 (8.3)	52 (86.7)
Type of medication history (Hx) previously taken asked	13 (21.7)	44 (73.3)
Presence of allergy history asked	0	57 (95)
Major adverse reaction (ADRs) asked	0	57 (95)
Exacerbating factors	2 (3.3)	55 (91.7)
Relieving factors	0	57 (95)

**Fig. 1:**
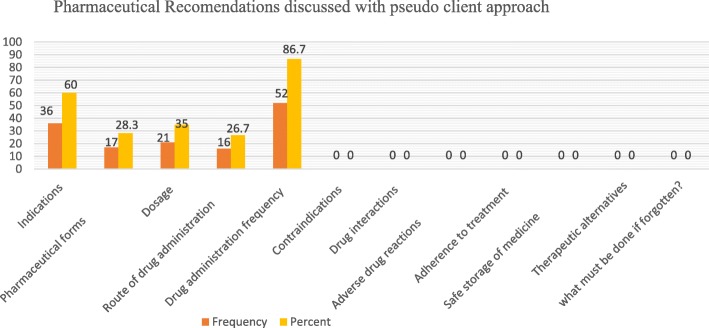
Pharmacotherapeutic recommendations dealt with during pseudo-client approach. Percentage and frequency for each Pharmacotherapeutic onsite recommendations forwarded by the Pharmacy professionals for the encountered pseudo clients

Among the most commonly signposted information that every dispensary personnel should counsel every patient when dispensing pharmaceutical formulations, drug administration times (frequency) (*n* = 52, 86.7%), medication indication (*n* = 36, 60%) and dosage form (*n* = 21, 35%) were the most discussed items during interaction with the simulated patients. To the contrary, none of the dispensers discussed important items such as contraindications, drug interactions, adverse drug reactions, adherence to treatment, and safe storage of the dispensed medications with the pseudo-clients (Fig. 1).

### Cross-sectional survey

Among the 60 dispensers that were approached, 51 of them completed and returned (response rate of 85%) the questionnaire. Almost two-thirds of the respondents were male (62.7%) and ages between 22 and 30 years old (72.5%). Of the CMROs, about 60% were leveled as pharmacies. A comparable number of pharmacists and druggists participated in the study. Average work experience as a dispenser in CMROLs was 5 years (±SD = 2.9). The majority (62.7%) of the dispensers reported to work for 8–10 h per day in the pharmacies with an average of 9.1 (±1.6) hours per day. Around 70% of participants reported that the average waiting time of clients in the dispensary was 5.2 (±2.34) minutes. Only one respondent had a guideline for headache management and 11.8% of the dispensers took clinical training in their work life. Detail socio-demographic characteristics are illustrated in Table 4.

**Table 4:** Socio-demographic characteristics of participants, (*N* = 51)

Characteristics	*N* (%)	Mean (±SD)
Sex		
Male	32 (62.7)	
Female	19 (37.3)	
Age in years		
22–30	37 (72.5)	29.7 (±4.1)
31–40	13 (25.5)	
>40	1 (2)	
Work experience in (years)		
<1 years	2 (3.9)	5 (±2.9)
1–5 years	32 (62.7)	
>5 years	17 (33.3)	
Length of working time (in hours)		
1–8 h	19 (37.3)	9.1 (±1.6)
8–10 h	32 (62.7)	
Average client waiting time (in minutes)		
1–5 min	35 (68.6)	5.2 (±2.34)
6–10 min	16 (31.4)	
Educational qualification		
Pharmacist	26 (51)	
Druggist	25 (49)	
Level of drug retail outlet		
Pharmacy	27 (52.9)	
Drug store	24 (47.1)	
Dispensers who had guideline for headache management		
Yes	1 (2)	
No	50 (98)	
Dispensers took clinical training in their work life		
Yes	6 (11.8)	
No	45 (88.2)	

According to the survey, most of the participants (64.7%) agreed or strongly agreed that managing headache symptomatically is challenging. However, many of them agreed or strongly agreed to the importance of syndrome-approach clinical training (52.9%) and continuous education and training (94.1%) to solve such challenges. In addition, a higher number of respondents (54.9%) agreed or strongly agreed that clients should involve in medication selection process (Table 5).

**Table 5:** Belief of dispensers on headache management challenges and solutions

Items	Number	Strongly disagree/Disagree (%)	Neutral (%)	Agree/strongly agree (%)
Managing headache symptomatically is challenging	51	9 (17.7)	9 (17.6)	33 (64.7)
Syndrome approach clinical training is important for treating headache	51	11 (21.5)	13 (25.5)	27 (52.9)
Continuous education and training improves challenges to treat headache	51	0	2 (3.9)	49 (94.1)
Patients should be involved in drug selection process	51	14 (27.4)	9 (17.6)	28 (54.9)

As to the respondents’ reports, only one-third (33.3%) of the clients had very good or excellent awareness with the dispenser’s role inregard to seeking additional information beyond what they have already obtained, and one-fourth (25.5%) were aware of generic and brand products (Table 6). The majority (66.7%) of dispensers reported that clients preferred brand products while other clients considered effectiveness (*n* = 17, 33.3%) and both price and effectiveness (*n* = 15, 29.4%) for choosing the medications. High proportions (78.4%) of community pharmacists and druggists recommended paracetamol for a non-examined headache, whereas, 60.8% of dispensers referred clients (who are taking paracetamol and in need of better treatment) to hospital or clinic. Less number of respondents recommended diclofenac (17.6%) and tramadol (13.7%). Lack of client interest towards professional counseling (41.2%) and lack of updated medical information provided by dispensers (31.4%) were the main potential barriers to counsel clients (Table 7).

**Table 6:** Community awareness’s and approaches towards CMROs

Items	Number	Poor/ fair (%)	Good (%)	Very good/ excellent (%)
Community awareness towards the role of community medicine retail outlets in headache management	51	5 (9.8)	29 (56.9)	17 (33.3)
Patients’ interest to get additional information beyond you provide	51	20 (39.2)	14 (27.5)	17 (33.3)
Communities awareness of generic and brand name of drugs	51	25 (50)	13 (25.5)	13 (25.5)

**Table 7:** Dispensers and clients drug selection and counseling barriers

Items	*N* (%)
Type of product preferred by clients	
Generic	17 (33.3)
Brand	34 (66.7)
What do client matters to choose their medication	
Price	5 (9.8)
Effectiveness	17 (33.3)
Price and effectiveness	15 (29.4)
Effectiveness and safety	10 (19.6)
Price, effectiveness and safety	4 (7.8)
Drugs recommended for non-examined headache	
Paracetamol	40 (78.4)
Diclofenac	5 (9.8)
Ibuprofen	3 (5.9)
Tramadol	3 (5.9)
Aspirin (ASA)	0
What would you do for a headache patient taking paracetamol who wanted better treatment	
Diclofenac	9 (17.6)
Ibuprofen	4 (7.8)
Tramadol	7 (13.7)
Refer to nearby hospital	31 (60.8)
Aspirin (ASA)	0
Potential barriers to counsel the clients	
High patient load	6 (11.8)
Shortage of time	3 (5.9)
Lack of un updated information	16 (31.4)
Patients lack of awareness to be counseled	5 (9.8)
Patient lack of interest	21 (41.2)

## Discussion

The present study evaluated the counseling manners and headache management practices of CMROs dispensers without a prescription in Gondar city. Essential variations concerning information provision and headache management practices of dispensers were discovered by comparing results found from the pseudo-client visits and the cross-sectional survey. Based on the SPs findings, the overall counseling approach was found to be 42.6%; however, it was improved to 58.3% when the pseudo-client demanded it. In the same way, findings in Riyadh, Saudi Arabia showed that the counseling level was found to be 43% even though it was enhanced when SPs demanded more information [28]. Depending on the type of investigation methodologies, the stated advising levels fluctuated from 8 to 100% in the worldwide literature [29]. Based on this, the reasons for such poor counseling practices might be multifactorial. The main challenges include lack of interest, poor experiences, knowledge and communication skills, and lack of standard counseling guideline.

In the real dispensing practices, 95% of dispensers provided medications for the pseudo-customers who were taking paracetamol while three dispensers advised the client to consult the physicians for identifying the cause of a headache without any further professional trail to help. This study is quite comparable with the study done in Saudi Arabia [28]. Contrarily, in the cross-sectional survey, 60.8% of participants referred the client to the hospital. This indicates that community pharmacists were not dispensing drugs based on knowledge and guidelines rather they sold for only cheesing money. Since most of CMROs are established for profit, no matter what the cause is, they sell every product without any hesitations. Moreover, unless they are profitable, their survival will be jeopardized and mainly unserved customers might disclose them to others clients that they do not serve well.

Concerning headache management using OTC medications, analgesics such as non-steroidal anti-inflammatory drugs (NSAIDs), acetaminophen, and weak opioids like tramadol are suggested as first-line drugs. Nevertheless, 64.7% of participants in the cross-sectional survey agreed that managing headache symptomatically is challenging, 78.4% of dispensers recommended acetaminophen for a non-examined headache, and about 40% of them added diclofenac, tramadol, and ibuprofen to paracetamol for better treatment. On the other hand, in the SPs approach, 95% of the participants primarily added Ibuprofen (45%), diclofenac (41.5%) and tramadol (5%) to acetaminophen for a headache management, though dispensers reported that customers chose brand products in the survey. Higher proportions of the dispensers selected the medications based on their effectiveness (61.7%) and cost (21.7%) which had similarities with survey results. In contrast, a pilot study in Brazil reported sodium dipyrone was the most recommended medication [30]. Even though customers do want brand products, suppliers rarely made them available for the community so that acetaminophen, ibuprofen, and diclofenac would be the first choice, since these drugs are safe, effective, readily available and affordable for most of the local customers.

During the pseudo-client approach, headache duration and signs or symptoms, medication profile and previous or current medical conditions were inquired by the dispensers. It is very important that dispensers pursue relevant evidence about clients’ history and compliant characteristic which enables them to choose appropriate pharmacotherapeutic alternatives for customers. However, forwarding many questions towards clients requires strong communication skills and knowledge, and increases client confidence and counseling satisfaction on dispensers. Such types of questions are highly supported by many comparative findings [31, 32].

Though higher proportions of participants (54.9%) in the cross-sectional survey agreed with the clients’ involvement of medication selection, smaller number of dispensers practically allowed the pseudo-client to be involved with their medication selection process. Surprisingly, nobody asked about whether the pseudo-client needed additional information, the presence of allergic history, adverse drug reaction history, and alleviating factors of a headache. Since self-medications are retailed without any prescription dispensers thought that many questions might discourage the patients form taking the medications [33]. Furthermore, in order to provide additional information and understand typical allergic history and adverse drug reactions providers should be trained for such types of evidence and clients’ interest. The survey revealed that clients’ absence of interest on dispensers and lack of updated medication information were found to be the major challenges for better counseling. However, 95% of respondents reported that the community was award of the role of community pharmacists in headache management.

When dispensing pharmaceutical formulations, there are important points that every dispenser should acknowledge and counsel the clients during providing drugs. However, the findings of this study revealed that only a few of the dispensers informed the clients about drug administration times (frequency), medication indication, dosage (strength), pharmaceutical forms, and route of drug administration during interaction with the simulated patients. To the contrary, none of the dispensers discussed important items such as contraindications, drug interactions, adverse drug reactions, adherence to treatment, and safe storage of the dispensed medications with the pseudo client. The findings of this study were similar to previous studies with regard to the rare provision of essential information by community pharmacists on precautions, adverse effects, drug interactions, contraindications, and safe storage [23, 24, 34]. But a Saudi study reported somewhat different results where 97% of the SPs visits provided information about dose, whereas a very small number of SPs were counseled on precaution. To the contrary, about half of the respondents never counseled on the side effects and drug interactions [28]. As explained earlier most of the provided counseling was superficial, easy and common that any health professionals might provide for every client. Mainly dispensers merely focus particularly on sales rather than counseling, because detailed discussions need further professional skills and extensive knowledge to deliver. Moreover, most of the patients do not seek more detailed information. Rather they need only what they want to know.

## Strength and limitations of the study

Every pseudo-client visit was audio-recorded to reduce the challenges associated with the human cognitive processes in conducting SP studies. After all, this study showed the real gap between the practical services and the theoretical expectations of community pharmacies. However, we used a convenience sampling method in only Gondar city. Therefore, generalizations of the study findings to other regions and populations should be with caution as it might lead to under or over representations. In addition, because only a specific case scenario was employed that leads to specific replies, it may not comprehensively assess the professionals’ competency towards a headache management. Moreover, because of the pseudo-client visit and the cross-sectional survey was conducted at different times, the respondents might not be the same and the responses to the self-administered questionnaire depended on the respondents’ trustworthiness which is subjected to socially desirable responses.

## Conclusions

Community pharmacists, being one of the most easily accessible health professionals in the community, are uniquely positioned to treat and manage minor ailments such as headache in their practice areas. According to findings from this study, community pharmacies demonstrated a very poor and inadequate skill headache management. Providing continuous clinical training and educational interventions are needed in order to mitigate the knowledge and skill gap. One suggestion is providing a hands-on evidence-based summary of headache management in their practice areas by academic institution and other stakeholders. Large scale studies that can explore community pharmacists’ involvement in managing headache in community pharmacies and that further could assess clients’ (purchasers’) level of satisfaction towards the community pharmacy service particularly regarding headache management is recommended to identify practice barriers and to better inform regulatory bodies.

## Abbreviations

ADR: Adverse Drug reaction
CMROs: Community medicine retail outlets
FMHACA: Food, Medicine, Healthcare Administration and Control Authority
GBD: Global Burden of Disease
Hx: History
MOH: Ministry of health
NSAIDs: Nonsteroidal anti-inflammatory drugs
OTC: Over the counter
PCM: Paracetamol
PCs: Pseudo clients
SD: Standard Deviation
SPs: Simulated patients
SPSS: Statistical Package of Social Sciences
TTH: Tension type headache
UK: United Kingdom
USD: United States Dollar
